# Few alterations in clinical pathology and histopathology observed in a CYP2C18&19 humanized mice model

**DOI:** 10.1186/1751-0147-50-47

**Published:** 2008-11-27

**Authors:** Susanne Löfgren, Stina Ekman, Ylva Terelius, Ronny Fransson-Steen

**Affiliations:** 1 Safety Assessment Sweden, AstraZeneca R&D Södertälje, S-151 85 Södertälje, Sweden; 2 Department of Biomedical Sciences and Veterinary Public Health, Division of Pathology, Pharmacology & Toxicology, Box 7028, SLU, S-750 07 Uppsala, Sweden; 3 Medivir AB, P.O. Box 1086, S-141 22 Huddinge, Sweden

## Abstract

**Background:**

This study was performed to characterize a gene-addition transgenic mouse containing a BAC (bacterial artificial chromosome) clone spanning the human CYP2C18&19 genes (tg-CYP2C18&19).

**Methods:**

Hemizygous tg-CYP2C18&19, 11 week old mice were compared with wild-type littermates to obtain information regarding clinical status, clinical pathology and anatomical pathology. After one week of clinical observations, blood samples were collected, organs weighed, and tissues collected for histopathology.

**Results:**

In males, the tissue weights were lower in tg-CYP2C18&19 than in wild-type mice for brain (*p *≤ 0.05), adrenal glands (*p *≤ 0.05) and brown fat deposits (*p *≤ 0.001) while the heart weight was higher (*p *≤ 0.001). In female tg-CYP2C18&19, the tissue weights were lower for brain (*p *≤ 0.001) and spleen (*p *≤ 0.001) compared to wild-type females. Male tg-CYP2C18&19 had increased blood glucose levels (*p *≤ 0.01) while females had decreased blood triglyceride levels (*p *≤ 0.01).

**Conclusion:**

Despite the observed alterations, tg-CYP2C18&19 did not show any macroscopic or microscopic pathology at the examined age. Hence, these hemizygous transgenic mice were considered to be viable and healthy animals.

## Background

The human cytochrome P450 enzymes from the 2C subfamily (CYP2C) are fairly well characterized and are known to metabolise many clinically important drugs. Four members belonging to the CYP2C family are found in man, namely CYP2C8, CYP2C9, CYP2C18 and CYP2C19 [1]. The anticancer drug paclitaxel is metabolised by CYP2C8 and the 6-hydroxylation of this compound is commonly used as a marker for this enzyme [2]. CYP2C9 metabolises many drugs, for example the hypoglycaemic drug tolbutamide [3], the anticonvulsant phenytoin [3,4], the anticoagulant warfarin [5] and a number of nonsteroidal anti-inflammatory drugs including diclofenac and ibuprofen [6], which have all been used as marker substrates. The CYP2C18 protein has not yet been found in detectable amounts in any tissues [7], and its *in vivo *function is, to date, unknown. CYP2C19 stereo-selectively metabolises the *S*-enantiomer of the anticonvulsant mephenytoin to the metabolite 4-hydroxy- (*S*)-mephenytoin [8], and this metabolite is commonly measured to determine CYP2C19 activity *in vitro*. The 5-hydroxylation of *R*-omeprazole is selectively performed by CYP2C19 [9] and this reaction is also occasionally used as marker reaction for CYP2C19. A variety of other substrates are known to be metabolised by CYP2C19, such as the biguanide antimalarials [10], certain barbiturates [11], the β-blocker propranolol [12], the anxiolytic diazepam [13] and the antidepressant imipramine [14].

In contrast to the relatively small human CYP2C family, the mouse Cyp2c family is one of the largest and most complex, with 15 members published to date [1](for an update, see ). Cyp2c29 was the first mouse Cyp2c member identified [15], followed by Cyp2c37, Cyp2c38, Cyp2c39, Cyp2c40 [16], Cyp2c44 [17], Cyp2c50, Cyp2c54 and Cyp2c55 [18]. Six additional murine Cyp2c enzymes have thereafter been identified; Cyp2c65, Cyp2c66, Cyp2c67, Cyp2c68, Cyp2c69 and Cyp2c70 [1]. Their metabolic preferences are poorly characterized but their organ distribution is partially known [16-19].

Murine CYP2C enzymes are involved in the metabolism of arachidonic acid, but the products formed differ between the isoforms. Human CYP2C19, on the other hand, is inhibited by the presence of arachidonic acid [20].

The transgenic mouse presented in this paper contains, in addition to all mouse Cyp2c enzymes, human CYP2C18 and CYP2C19. The inserted human *CYP2C18 *and *CYP2C19 *genes are expressed at the mRNA and protein levels, and the inserted *CYP2C19 *genes have been shown to be functional *in vitro *in metabolism studies using the CYP2C19 substrates *S*-mephenytoin and *R*-omeprazole)[21].

The aim of the present study was to characterize the humanized CYP2C18&19 mouse model as a basis for upcoming pharmacokinetic and toxicological studies. Hemizygous humanized CYP2C18&19 mice (tg-CYP2C18&19) were compared with wild-type littermates to obtain information regarding clinical status, body weight, clinical pathology, anatomy and morphology of this particular mouse model.

## Methods

### Generation of BAC transgenic mice

The transgenic CYP2C18&19 mice characterized in this article were generated as previously described)[21]. In brief, a BAC (bacterial artificial chromosome) clone named BAC RP11-466J14, which contains the *CYP2C18 *and *CYP2C19 *genes was purified. BAC DNA was injected into C57BL/6JOlaHsd (C57BL/6) eggs. Founders were identified by genotyping of DNA extracted from tail or ear biopsies.

### Genotyping

For PCR detection of the inserted gene segment, gDNA was extracted from tail or ear biopsies either by using established protocols [22] or commercially available kits (DNeasy^® ^Tissues, Qiagen). The gDNA was amplified in a 20 μL reaction mixture containing 10 μL HiFi PCR MasterMix (ABgene House, Surrey, UK), primers (250 nM of each primer for males or, alternatively, 500 nM of each primer for females) and 1 μL of gDNA. The four different specific primer pairs used are listed in Table 1. Cycling conditions were 94°C for 2 minutes (denaturation) and then 30 cycles of 94°C for 10 seconds, 60°C for 20 seconds, and 68°C for 45 seconds, followed by a 3 minutes extension at 70°C. The amplification products were analyzed on 1% agarose gels and the amplicons visualized with ultraviolet light.

**Table 1:** Sequences of primers used for genotyping of the mice (to detect the 466J14 BAC clone containing human CYP2C18 and CYP2C19)

**Primer**	**Primer sequence (5'-3')**	**PCR product length (bp)**
BAC5'endF	TAACATTAGCAGGTGAAGCCCAAA	706
BAC5'endR	CAATCTGTTCCATGATGGTTGATG	
BAC3'endF	AGACTGTGCTATCATGGGAACCAA	480
BAC3'endR	GTTTTCTTGGGCTGAATGTCCTCT	
2C18intron6F	GGCAAGAAACACTTCATGAGCACT	429
2C18intron6R	ATTCAGTTAAGGCCTCCCTTTTCC	
2C19intron5F	CAAGATGGGCCTTATAAAGTTGGC	727
2C19intron5R	GAAGAAATTGGAACCCTCATGTCC	

### Animal husbandry

The hemizygous tg-CYP2C18&19 and the C57BL/6JOlaHsd (C57BL/6) wild-type littermates used were generated by crossing hemizygous tg-CYP2C18&19 males with C57BL/6JOlaHsd (C57BL/6) wild-type female mice. Wild-type littermates were used as controls. Attempts were also performed to generate homozygous mice by crossing hemizygous tg-CYP2C18&19 males and females, but the offspring died within a few days from birth.

For logistic reasons, the male and female groups were housed at different sites, but all mice were kept under conventional conditions and had free access to standard rodent diet (Males: R&M 1.E. SQC, pelleted diet, supplied by Special Diets Services Ltd, England; Females: RM3 Extended Breeding, supplied by Special Diets Services Ltd, England) and tap water. The animal husbandry and experimental conditions were approved by the Swedish Animal Welfare Agency.

### Analysis of weight gain and food consumption

All mice were observed for one week prior to necropsy at an age of approximately 11 weeks. During that week, any adverse clinical signs observed on ocular inspection were recorded and the body weight gain and food consumption were measured. Individual body weights were recorded four and six days before necropsy for males and three and six days before necropsy for females. Six males and six females from each genotype (wild-type and tg-CYP2C18&19) were examined.

### Pathological evaluation, total body weight and tissue weights

The total body weight of each mouse was determined, to the nearest 0.1 g, prior to necropsy and animals were killed by exsanguination of the common carotid artery under enflurane and nitric oxide anaesthesia. During necropsy, the organs were examined macroscopically and weights of a standard set of tissues (Table 2) were measured, to the nearest mg, prior to fixation. For bilateral organs, the total weight of the pair was recorded.

**Table 2:** Tissues sampled at necropsy

**Tissue**	**Weighed at necropsy**	**Tissue**	**Weighed at necropsy**
Adrenal glands	Yes	Muscle-skeletal	
Aorta (thoracic)		Nerve-sciatic	
Bone and bone marrow (sternum)		Optic nerves^a^	
Brain	Yes	Ovaries	Yes
Brown fat deposit^a^	Yes	Pancreas	
Cervix		Parathyroid glands^b^	
Epididymides		Pituitary gland	
Epididymal fat deposit	Yes	Prostate gland-ventral	Yes
Oesophagus^a^		Retriperitoneal fat deposit	Yes
Eyes		Salivary gland-parotid	
Femur/femoro-tibial joint^a^		Salivary gland-submaxillary/lingual	
Harderian gland^a^		Seminal vesicles	
Heart	Yes	Skin	
Intestine-duodenum		Spleen^a^	Yes
Intestine-jejunum		Spinal cord-lumbar and cervical	
Intestine-ileum		Stomach	
Intestine-colon		Testes	Yes
Intestine-caecum		Thymus	Yes
Intestine-rectum		Thyroid glands^b^	
Kidneys	Yes	Tongue	
Liver with gallbladder	Yes	Trachea	
Lungs	Yes	Urinary bladder	
Lymph node-mandibular		Uterus	Yes
Lymph node-mesenteric^a^		Vagina	

a/Tissue lost during processing in 1–2 animals and was not evaluated histologically.

b/Tissue lost during processing in all females but one per genotype. The tissue was present on slides from all male mice.

Forty-eight tissues (Table 2) from each mouse were collected and fixed. Eyes were fixed in MFAA (Methanol, Formalin, Acetic Acid); testicles and epididymides were fixed in Bouin's solution and all other tissues in 4% buffered formaldehyde. All tissues preserved were dehydrated, embedded in paraffin and cut into 4 μm sections before they were stained with haematoxylin and eosin for microscopic evaluation.

### Clinical pathology parameters and analytical methods

Blood samples for haematology (EDTA tubes) and blood chemistry (lithium heparin tubes) were collected from the orbital plexus under enflurane and nitric oxide anaesthesia, prior to necropsy. Animals were not fasted at the time of blood sampling but the genotype groups were necropsied with every second animal being wild-type and every second being transgenic in order to minimize daytime variations in glycogen content between the groups. At necropsy, one femur was taken for bone marrow analysis. Haematology analysis was performed with an ADVIA^® ^120 Haematology System (Bayer Corporation, Diagnostic Division, Tarrytown, US) using standard methodology. Blood chemistry parameters were analysed using a Cobas Integra 400 analyser (Roche Diagnostics Instrument Centre, Switzerland), and appropriate kits. Parameters measured in haematology and blood chemistry are listed in Table 3.

**Table 3:** Parameters measured in clinical pathology

**Haematology**	**Blood chemistry**
Basophils (Baso)	Albumin (Alb)
Erythrocytes (RBC)	Albumin/globulin ratio (A/G)
Eosinophils (Eosn)	Alkaline aminotransferase (ALT)
Hematocrit (Hct)	Alkaline phosphatase (ALP)
Haemoglobin (Hgb)	Aspartate aminotransferase (AST)
Large unstained cells (LUC)	Bilirubin (total) (Bil)
Leucocytes (WBC)	Calcium (Ca)
Lymphocytes (Lymp)	Cholesterol (Chol)
Mean corpuscular haemoglobin (MCH)	Creatinine (Cre)
Mean corpuscular haemoglobin concentration (MCHC)	Glucose (Glu)
Mean red cell volume (MCV)	Potassium (K)
Monocytes (Mono)	Sodium (Na)
Neutrophils (Neut)	Total protein (TP)
Platelets (Plt)	Triglycerides (TG)
Red cell distribution width (RDW)	Urea (Urea)
Reticulocytes (Retc)	

The bone marrow differentials were determined by flow cytometry as described by Saad et al [23] and the total nucleated cell count, the myeloid: erythroid ratio and the proportion of lymphoid, myeloid, erythroid and nucleated cells were determined. In addition, the proportion of cells staining positive for LDS-751 (laser dye styryl-751) was determined.

### Statistical analysis

The statistical comparisons between the transgenic and wild-type groups were performed, using a computerized statistical program (Sigma Stat, version 2.03). All variables were compared with a t-test. Organ weights were compared both as absolute weights and as relative weights (relative to brain and relative to total body weight, data not shown). Box plots in figures were generated using Sigma Plot, 2001 and the plots show the median (line within each box), the 25^th^/75^th ^percentile (outer boundaries of each box) and the 10^th^/90^th ^percentiles (whiskers above and below each box). In addition the minimum and maximum are marked with dots.

## Results

### Weight gain and food consumption

All male mice either retained or increased their weight during the *in vivo *part of the study. In the female groups most of the mice gained weight. Two wild-type females and two tg-CYP2C18&19 females decreased in weight, but the weight loss in all cases was ≤ 0.6 g. No significant differences in food consumption were recorded.

### Pathological evaluation

At necropsy, all mice were in good nutritional condition. On macroscopical examination, small white foci were found in the eyes of one wild-type female and two tg-CYP2C18&19 females. These macroscopical changes, and the recorded weight differences between groups, did not correlate to any changes on the histopathological level. Minimal perivascular infiltration of neutrophils was found on microscopic examination in the epididymal fat in one tg-CYP2C18&19 male and minimal alveolar histiocytosis was present in one tg-CYP2C18&19 female. All these changes were considered to belong to the spontaneous background pathology observed in laboratory mice of the C57BL/6JOlaHsd strain.

### Total body weight and tissue weights

**Figure 1:**
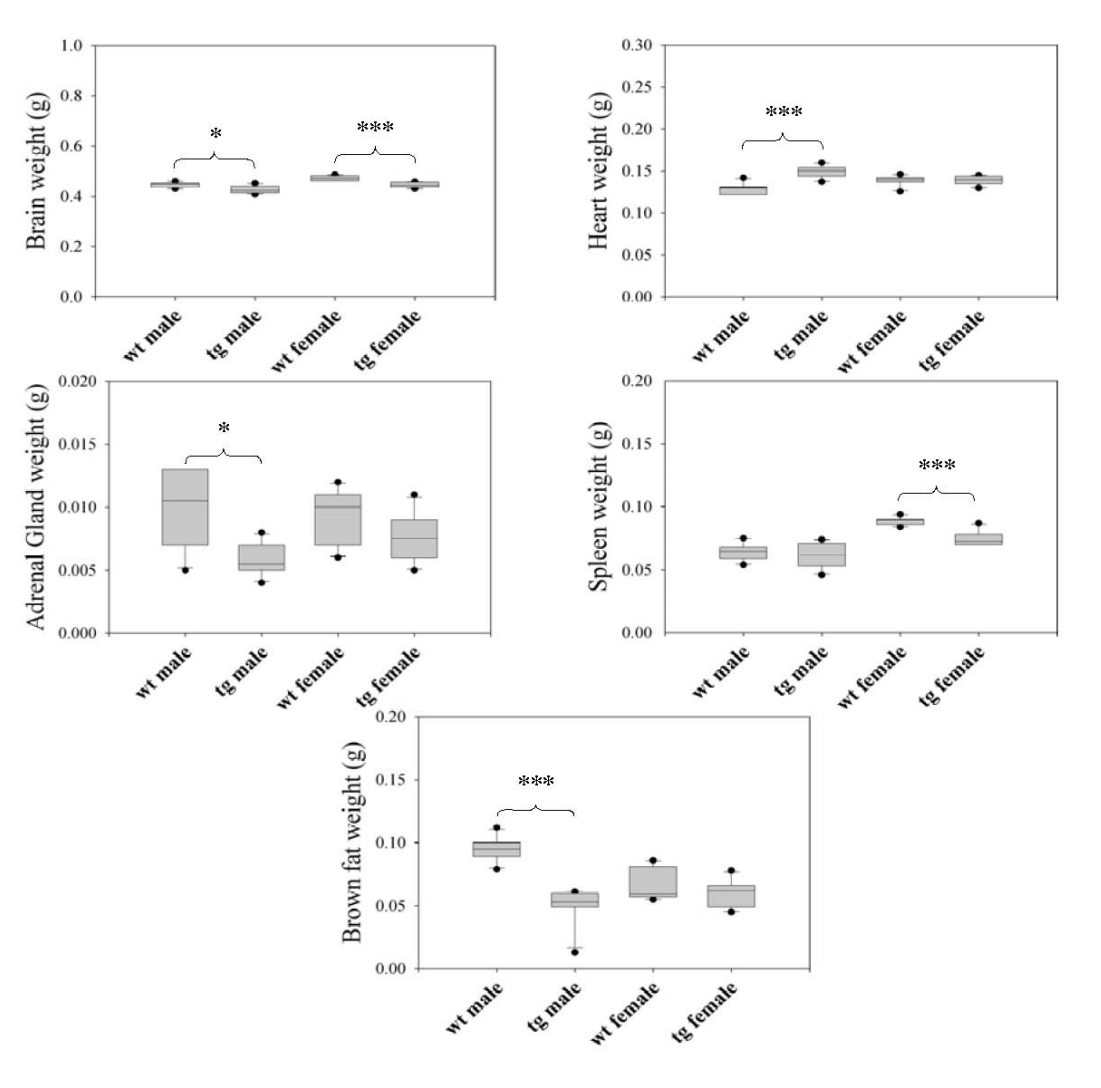
**Comparison of tissue weights between CYP2C18&19 transgenic and wild-type mice**. The figure shows the absolute tissue weights for tissues with statistically significant differences between genotypes. Each group contains 6 animals. Asterisks indicate significant differences between groups, *p ≤ 0.05, ***p ≤ 0.001. wt: wild-type C57BL/6 mice, tg: hemizygous transgenic mice containing human CYP2C18&19. The box plots are presented as described in materials and methods.

Weight distributions for all tissues within the different genotype and sex groups are shown in Table 4 and the statistically significant organ weight alterations are shown in Figure 1. Organ weights were compared between genotypes, both as absolute weights, relative to brain weight and relative to the total body weight. The relative data are not presented, since the same organs showed statistically significant weight differences between genotypes regardless of which comparison was used. The only exception was the brain weight relative to total body weight in male mice, which did not show any statistical difference between the transgenic and the wild-type groups.

**Table 4:** A comparison of absolute tissue weights between CYP2C18&19 transgenic and wild-type mice

	♂		♀	
Tissue	Wt	Tg	Wt	Tg
Body (g)	22.3 ± 1.5	22.2 ± 1.3	21.4 ± 0.5	21.0 ± 0.6
Brain (mg)	446 ± 11.4	427 ± 16.6*	472 ± 9.93	444 ± 11.9***
Heart (mg)	130 ± 7.39	149 ± 8.06***	139 ± 6.80	139 ± 6.05
Lung (mg)	148 ± 15.4	145 ± 5.61	159 ± 12.8	157 ± 18.9
Liver (mg)	1286 ± 76.2	1267 ± 102	1325 ± 89.7	1271 ± 54.2
Kidney (mg)	291 ± 18.9	286 ± 18.8	328 ± 9.51	315 ± 12.9
Adrenal glands (mg)	9.83 ± 3.25	5.83 ± 1.47*	9.33 ± 2.42	7.67 ± 2.16
Spleen (mg)	64.2 ± 7.41	61.0 ± 10.6	88.8 ± 3.49	75.0 ± 6.75***
Thymus (mg)	41.2 ± 7.31	37.7 ± 13.2	60.5 ± 7.45	68.0 ± 11.1
Retriperitoneal fat (mg)	69.7 ± 25.9	61.5 ± 8.07	25.8 ± 11.5	26.3 ± 7.61
Brown fat (mg)	95.0 ± 11.0	48.2 ± 17.9***	66.3 ± 13.5	60.2 ± 12.3
Testes (mg)	209 ± 19.3	210 ± 12.1		
Prostate (mg)	39.3 ± 12.5	49.2 ± 9.11		
Epididymal fat (mg)	306 ± 80.0	287 ± 37.0		
Uterus (mg)			89.5 ± 43.0	112 ± 47.0
Ovaries (mg)			10.8 ± 6.68	13.3 ± 6.35

The table shows the average absolute tissue weights for each group +/- standard deviations. The statistical comparisons were performed as described in materials and methods. *p ≤ 0.05, ***p ≤ 0.001. wt: wild-type C57BL/6 mice, Tg: hemizygous transgenic mice containing human CYP2C18&19.

In both male and female mice, the brain weight was lower in tg-CYP2C18&19 than in wild-type mice (*p *≤ 0.05 and *p *≤ 0.001 for males and females respectively). The adrenal glands (*p *≤ 0.05) and brown fat deposits (*p *≤ 0.001) were smaller, while the heart weight was larger (*p *≤ 0.001) in the tg-CYP2C18&19 males than in wild-type males. The spleen weight was lower in female tg-CYP2C18&19 than in wild-typefemales (*p *≤ 0.05). All other organ weight comparisons (lung, liver, kidney, thymus, retriperitoneal fat deposits, testis, prostate, epididymal fat deposits, uterus and ovaries) showed no significant differences between the genotypes.

### Clinical pathology parameters

**Figure 2:**
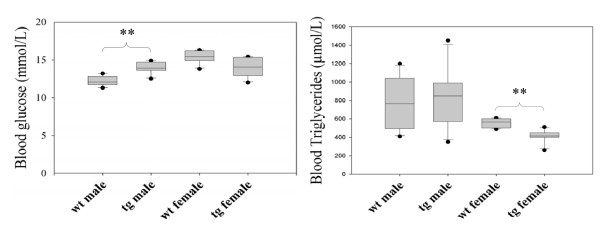
**Comparisons of clinical pathology parameters between CYP2C18&19 transgenic and wild-type mice**. The figure shows the blood chemistry parameters with statistically significant differences between genotypes. Each group contains 6 animals. Asterisks indicate significant differences between groups, **p ≤ 0.01. wt: wild-type C57BL/6 mice, tg: hemizygous transgenic mice containing human CYP2C18&19. The box plots are presented as described in materials and methods.

Comparisons of all clinical pathology variables (between the genetic and sex groups) are shown in Table 5. Clinical pathology parameters with significant differences (*p *≤ 0.05) between the genotypes are shown in Figure 2.

**Table 5:** Comparisons of haematology and blood chemistry parameters between genotypes of both sexes

Haematology parameter	♂		♀		Blood chemistry parameter	♂		♀	
	Wt	Tg	Wt	Tg		Wt	Tg	Wt	Tg
RBC (*10^12^/L)	8.89 ± 0.213	8.72 ± 0.200	9.78 ± 0.496	9.54 ± 0.286	GLU (mmol/L)	12.2 ± 0.708	13.9 ± 0.88**	15.3 ± 0.97	14.0 ± 1.33
HGB (g/L)	136 ± 1.51	134 ± 1.97	148 ± 8.80	146 ± 4.90	UREA (mmol/L)	7.10 ± 1.58	6.62 ± 1.20	8.58 ± 1.16	8.03 ± 1.33
Hct (%)	43.0 ± 0.894	42.3 ± 0.816	48.0 ± 1.79	47.3 ± 1.21	TP (g/L)	49.2 ± 1.47	48.2 ± 0.75	46.3 ± 1.21	45.7 ± 1.21
MCV (fL)	48.5 ± 1.18	48.7 ± 0.853	49.2 ± 0.809	49.6 ± 0.501	ALB (g/L)	28.0 ± 1.27	27.5 ± 0.548	29.8 ± 0.983	29.5 ± 1.23
MCH (pg)	15.3 ± 0.273	15.4 ± 0.303	15.1 ± 0.293	15.3 ± 0.341	A/G ratio	1.33 ± 0.121	1.33 ± 0.0516	1.80 ± 0.0632	1.85 ± 0.176
MCHC (g/L)	316 ± 4.50	316 ± 4.41	307 ± 8.31	308 ± 6.05	Na (mmol/L)	148 ± 1.03	147 ± 1.38	146 ± 0.516	147 ± 0.837
PLT (*10^12^/L)	1.18 ± 0.0394	1.18 ± 0.0299	1.04 ± 0.0714	1.07 ± 0.0659	K (mmol/L)	4.42 ± 0.475	4.32 ± 0.313	3.90 ± 0.329	3.83 ± 0.344
WBC (*10^9^/L)	5.21 ± 1.95	4.37 ± 1.05	2.84 ± 1.28	2.32 ± 0.792	ALT (U/L)	79.2 ± 25.4	63.2 ± 18.9	60.3 ± 18.3	45.0 ± 3.35
Neutr (*10^6^/L)	823 ± 752	470 ± 220	597 ± 646	483 ± 402	AST (U/L)	108 ± 62.1	77.0 ± 18.9	110 ± 58.5	93.5 ± 34.2
Lymp (*10^9^/L)	3.99 ± 1.46	3.65 ± 0.975	2.04 ± 0.972	1.71 ± 0.589	ALP (U/L)	111 ± 8.98	113 ± 11.3	122 ± 13.6	134 ± 16.2
Mono (*10^6^/L)	260 ± 237	86.7 ± 24.2	137 ± 95.8	66.7 ± 30.1	TBil (μmol/L)	7.66 ± 1.97	6.83 ± 1.33	11.7 ± 3.33	10.2 ± 1.72
Eosn (*10^6^/L)	107 ± 35.0	127 ± 62.8	63.3 ± 55.7	46.7 ± 35.0	Crea (μmol/L)	8.33 ± 1.86	8.50 ± 1.52	7.83 ± 1.47	7.00 ± 0.894
Baso (*10^6^/L)	6.67 ± 10.3	3.33 ± 8.16	10.0 ± 16.7	0 ± 0	Ca (mmol/L)	2.50 ± 0.0565	2.50 ± 0.0446	2.37 ± 0.0681	2.34 ± 0.0186
LUC (*10^6^/L)	30.0 ± 21.0	30.0 ± 11.0	6.67 ± 10.3	0 ± 0	Chol (mmol/L)	2.42 ± 0.117	2.43 ± 0.163	1.70 ± 0.179	1.67 ± 0.197
Reti (*10^9^/L)	283 ± 27.5	288 ± 15.1	271 ± 36.7	295 ± 30.7	TG (μmol/L)	795 ± 327	848 ± 372	555 ± 50.9	408 ± 83.3**
RDV (%)	12.6 ± 0.147	12.6 ± 0.261	12.9 ± 0.388	12.6 ± 0.210					

The table shows the average haematology and blood chemistry values for each group +/- standard deviations. The statistical comparisons were performed as described in materials and methods, and the abbreviations used are explained in Table 3. **p ≤ 0.01.

wt: wild-type C57BL/6 mice, Tg: hemizygous transgenic mice containing human CYP2C18&19.

The blood glucose levels were altered in male tg-CYP2C18&19 mice, which had higher levels than wild-type males (*p *≤ 0.01). The level of circulating triglycerides in the blood was decreased in female tg-CYP2C18&19 compared to the wild-type females (*p *≤ 0.01).

All other haematology parameters and blood chemistry parameters measured showed no significant differences between the genotypes (See Table 3). There were no significant differences between the genotypes in any of the bone marrow parameters measured (total nucleated cell count, myeloid: erythroid ratio and the proportion of lymphoid, myeloid, erythroid and nucleated cells). The bone marrow parameter results are shown in Table 6.

**Table 6:** Comparisons of bone marrow parameters between genotypes of both sexes

Bone marrow parameter	♂		♀	
	Wt	Tg	Wt	Tg
TNC (*10^6^/femur)	8.78 ± 0.995	9.27 ± 0.726	14.4 ± 2.71	11.7 ± 1.35
% Erythroid	37.8 ± 2.89	38.2 ± 3.51	33.6 ± 3.76	34.8 ± 5.88
% Lymphoid	12.0 ± 1.55	11.0 ± 1.40	8.41 ± 1.70	10.2 ± 1.48
% Myeloid	50.1 ± 3.38	50.6 ± 3.91	57.7 ± 4.81	54.6 ± 5.55
Ratio M: E	1.34 ± 0.188	1.35 ± 0.259	1.75 ± 0.342	1.64 ± 0.500
% LDS+	85.2 ± 2.19	85.7 ± 3.01	90.1 ± 2.60	89.4 ± 1.54

The table shows the average bone marrow values for each group +/- standard deviations. The statistical comparisons were performed as described in materials and methods.

wt: wild-type C57BL/6 mice, Tg: hemizygous transgenic mice containing human CYP2C18&19. TNC: Total Nucleated Count, % Erythroid/Lymphoid/Myeloid: Proportion (of total)

Erythroid/Lymphoid/Myeloid cells, Ration M: E: Myeloid: Erythroid ratio, %LDS+: percentage of (total) cells staining positive for the nucleic acid stain LDC-751 (laser dye styryl-751).

## Discussion

The regulation and functions of the members of the CYP2C family are complex with large variations in expression within, and between, different tissues. The humanized mouse model characterized in this paper may facilitate the study and understanding of the functions of the human CYP2C enzymes. The demonstrated alterations in organ weights and clinical chemistry parameters cannot all be explained with the current knowledge of the CYP2C enzymes.

Arachidonic acid is metabolised in human brain parenchymal tissue to epoxyeicosatrienoic acid, which acts as a potent dilator of cerebral vessels [24]. In rats, this metabolism is carried out by CYP2C11 in the brain [25] and it is therefore possible that other members of the CYP2C subfamily, together with members of other cytochrome P450s subfamilies present in astrocytes, also participate in the metabolism of arachidonic acid in other species. The mouse Cyp2c enzymes are also involved in the metabolism of arachidonic acid and the murine isoforms metabolize arachidonic acid to regio- and stereospecific products[18]. This metabolism could be altered by the insertion of human CYP2C18&19 genes in the mouse model presented and, thereby, influence cerebral blood flow and possibly also the brain weight. The activities of the CYP2C enzymes in the central nervous system have also been proposed to influence the action of neurotransmitters, such as dopamine, which utilize fatty acid metabolites as intracellular mediators [26].

Alterations in the metabolism of arachidonic acid could possibly also explain the increased heart weight in the male tg-CYP2C18&19 mice. The CYP2C enzymes expressed in the cardiovascular system play a crucial role in the modulation of vascular homeostasis [27]. CYP products such as epoxyeicosatrienoic acids and reactive oxygen species have been implicated in the regulation of intracellular signalling cascades and vascular cell proliferation [28]. Preliminary behavioural studies show an initial increase in locomotor activity for male tg-CYP2C18&19 mice compared to wild-type controls when the mice are put in activity boxes. The increased activity of the transgenic mice could possibly also contribute to the increased heart weight (data not shown).

When focusing on lipid and glucose metabolism, the interactions are even more complex. In the present study, male tg-CYP2C18&19 mice had decreased brown fat deposits compared to wild-type mice and female tg-CYP2C18&19 had decreased levels of circulating triglycerides. The glucose levels were increased in male tg-CYP2C18&19 mice compared to wild-type males. Exogenous glucose administration to rats has been shown to decrease CYP2C6 and the male specific CYP2C11 activity by altering hepatic lipids [29]. If a similar male specific regulation occurs in the tg-CYP2C18&19 mice, this could possibly explain the alterations in glucose levels, fat deposits and blood triglyceride levels observed in this study.

Despite the few organ weight and clinical chemistry alterations observed, the hemizygous tg-CYP2C18&19 mice are considered to be viable and healthy. The alterations observed are also unlikely to cause any decrease in lifespan of the strain since the few tg-CYP2C18&19 male mice kept as breeders have reached an age of 2–3 years (unpublished data).

## Conclusion

In the present study a gene-addition transgenic mouse, containing a BAC spanning the human CYP2C18&19 genes, has been characterized. Some alterations in organ weight and clinical pathology parameters were observed. Despite the alterations, no pathological changes were observed macroscopically or histologically and these hemizygous tg-CYP2C18&19 mice were considered to be viable and healthy. Hopefully, this model could be used to investigate the roles of CYP2C18 and CYP2C19 *in vivo *and extrapolation of results obtained from studies with this model may be more predictive to humans than when using traditional animal models.

## Competing interests

The authors declare that they have no competing interests.

## Authors' contributions

SL carried out the pathological evaluation of the study, participated in the design of the study, performed the statistical analysis and drafted the manuscript. SE and YT helped to draft the manuscript. R F-S helped to draft the manuscript and funded the study. SE, YT and R F-S have all been supervisors to SL during the course of the study.
